# Chitosan Nanoparticles for Gastroesophageal Reflux Disease Treatment

**DOI:** 10.3390/polym15163485

**Published:** 2023-08-20

**Authors:** Yedi Herdiana

**Affiliations:** Department of Pharmaceutics and Pharmaceutical Technology, Faculty of Pharmacy, Universitas Padjadjaran, Sumedang 45363, Indonesia; y.herdiana@unpad.ac.id

**Keywords:** chitosan nanoparticles, gastroesophageal reflux disease, GERD treatment, nanotechnology, targeted drug delivery, esophageal tissue protection

## Abstract

Gastroesophageal Reflux Disease (GERD) is a chronic ailment that results from the backward flow of stomach acid into the esophagus, causing heartburn and acid regurgitation. This review explores nanotechnology as a novel treatment approach for GERD. Chitosan nanoparticles (CSNPs) offer several advantages, including biocompatibility, biodegradability, and targeted drug delivery capabilities. CSNPs have been extensively studied due to their ability to encapsulate and release medications in a controlled manner. Different nanoparticle (NP) delivery systems, including gels, microspheres, and coatings, have been developed to enhance drug retention, drug targeting, and controlled release in the esophagus. These nanoparticles can target specific molecular pathways associated with acid regulation, esophageal tissue protection, and inflammation modulation. However, the optimization of nanoparticle formulations faces challenges, including ensuring stability, scalability, and regulatory compliance. The future may see CSNPs combined with other treatments like proton pump inhibitors (PPIs) or mucosal protectants for a synergistic therapeutic approach. Thus, CSNPs provide exciting opportunities for novel GERD treatment strategies.

## 1. Introduction

GERD is a common digestive disorder that affects many people worldwide [1,2,3,4]. It’s becoming more common in high-income countries (15–25%) compared to low- and middle-income countries (<10%). GERD can greatly impact a person’s quality of life and requires long-term treatment, which can be expensive [5,6,7,8]. It is important to address GERD effectively to improve people’s well-being and manage healthcare costs. GERD is a chronic condition where stomach acid and digestive juices flow back into the esophagus or beyond (oral cavity, larynx, or the lungs), causing troublesome symptoms and complications. Symptoms include heartburn, acid regurgitation, chest pain, and difficulty swallowing [9]. The pathophysiology of GERD is multifactorial, and a stepwise approach will assist physicians in making the diagnosis. GERD is a primary risk factor for esophageal adenocarcinoma [10].

Several lifestyle-related and modifiable risk factors for GERD have been demonstrated. GERD was found to be more common in individuals who used non-steroidal anti-inflammatory drugs (NSAIDs) and individuals who were aged over 50, smokers, obese, with anxiety or depression, decreased physical activity, and consumption of fast food. Other factors include a possible role of high caffeine consumption and the decreased prevalence of *Helicobacter pylori* (*H. pylori*) infection in the region [9,11,12,13,14,15,16,17,18,19]. Recently, non-alcoholic fatty liver disease has been reported to increase the risk of developing reflux esophagitis. Besides environmental factors, genetic factors may contribute to the differences across different ethnic groups [19,20]. Eating habits such as irregular meal patterns, a large volume of meals, and eating meals just before bedtime may correlate with the symptoms of GERD [21]. Significant variations in GERD prevalence were found between regions and countries, and we have demonstrated that lifestyle, socioeconomic, and sociodemographic factors may contribute to these variations [22].

The diagnosis is commonly determined by considering a range of presenting symptoms, performing objective testing via endoscopy, monitoring reflux using ambulatory techniques, and assessing the response to PPI therapy. Medications that impact the pressure of the lower esophageal sphincter, such as nitrates, calcium channel blockers, and anticholinergics, also contribute to this diagnostic process [9]. Although several treatment options are available for GERD, such as lifestyle modifications, dietary changes, medications, and surgical interventions, there are still challenges in achieving optimal disease management. Some patients may experience incomplete symptom relief, side effects from medications, or the need for long-term maintenance therapy. Additionally, ensuring the targeted delivery of therapeutic agents to the affected areas in the gastrointestinal (GI) tract poses a challenge in GERD treatment.

As there is no universally accepted definitive method for diagnosing GERD, the diagnosis relies on a comprehensive assessment of symptom presentation, endoscopic examination of the esophageal mucosa, reflux monitoring, and evaluating the response to therapeutic interventions [23]. Pharmaceutical and surgical interventions have been devised to address GERD. However, pharmaceutical medications typically only relieve GERD symptoms and may carry the risk of significant side effects. On the other hand, surgical procedures are invasive. Consequently, exploring different endo-luminal outpatient therapies for GERD presents a more appealing alternative [24]. A thorough evaluation by a healthcare professional specializing in GERD management is necessary to determine the most appropriate treatment option for each patient.

Nanotechnology has surfaced as a compelling strategy in medicine, offering significant potential and new possibilities for targeted drug delivery and enhanced therapeutic efficacy [25,26]. In the context of GERD, NPs have shown the potential to address the challenges associated with conventional treatment methods [27,28,29]. By utilizing NPs, it becomes possible to enhance drug stability, prolong drug release, protect drugs from degradation in the harsh gastric environment, and specifically target the affected areas in the esophagus [29,30,31]. NPs in GERD management allow for precise control over drug release profiles, enabling sustained and localized drug delivery to the inflamed esophageal tissues. Moreover, NPs can undergo functionalization by adding targeting ligands or surface modifications to enhance their interaction with affected tissues and improve therapeutic efficacy.

This review focuses on the challenges in treating GERD and explores the potential of NPs as a promising solution. Specifically, it discusses the use of CSNPs for GERD treatment, covering different types of NPs, how they are formulated, their mechanisms of action, recent advancements, and future possibilities.

## 2. Gastroesophageal Reflux Disease (GERD)

### 2.1. General Overview of GERD

**Figure 1:**
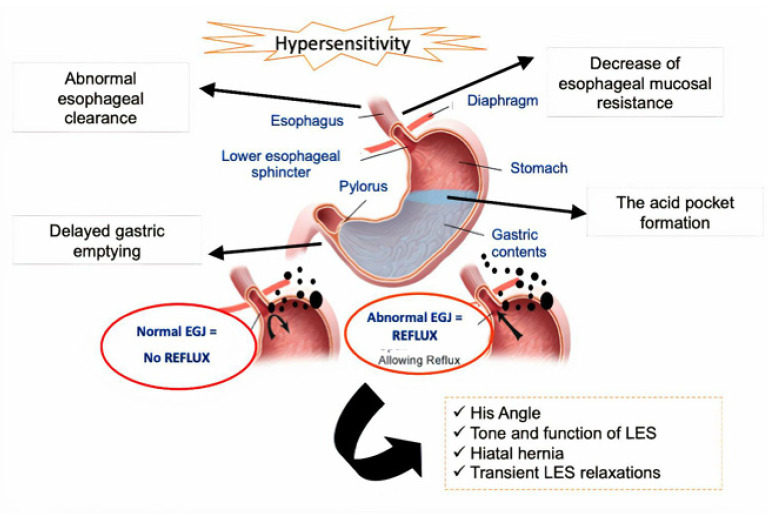
The complex pathogenesis of GERD. (Reprinted with permission from *Drug Design, Development Therapy* 2021:15 1609–1621, Dove Medical Press Ltd.) [12].

GERD, a condition characterized by recurrent and troublesome heartburn and regurgitation, affects approximately 20% of adults in high-income countries [14]. Its prevalence is notably high in Western countries, ranging from 13% to 20% in the USA and 9.8% to 18% in Europe, while Asia experiences lower rates at 2.5% to 4.8% [12]. Laryngopharyngeal reflux (LPR), considered an extraesophageal symptom of GERD, occurs when stomach contents flow backward into the esophagus, larynx, and pharynx, resulting in tissue damage and symptoms like odynophagia, pharyngeal globus, throat clearing, dysphonia, dry cough, and laryngospasm crisis [14]. GERD has also been linked to increased oral cavity acidity, which can lead to dental erosion. However, the relationship between GERD and periodontal disease remains controversial [13]. It is important to note that weakened GI sphincters, such as the one at the gastroesophageal junction, contribute to the retrograde flux of contents causing GER. In contrast, sphincters at the anal level can lead to fecal incontinence [32]. The complex pathogenesis of GERD is shown in Figure 1.

GERD primarily affects the lower esophageal sphincter and can be categorized into two types: non-erosive disease (NERD) and erosive reflux disease (ERD), depending on whether esophageal erosions are present during endoscopic examination [12,33]. While the disease has a higher incidence in men, it is more likely to manifest as NERD in women. Significant risk factors for GERD include obesity, advancing age, a family history of reflux disease, and prolonged use of specific medications such as nitrates, calcium antagonists, and benzodiazepines, among others [12].

A hiatal hernia exacerbates gastroesophageal reflux by intensifying the contact between stomach acid and the esophagus. It disrupts the interaction between the lower esophageal sphincter (LES) and the crural diaphragm, essential components of the anti-reflux barrier. This impairment in the coupling between the LES and the crural diaphragm contributes to the reflux of stomach contents into the esophagus. Impaired esophageal clearance prolongs the contact between refluxate and the esophageal mucosa, leading to symptoms and potential damage [34,35]. Esophageal motility disorders, delayed gastric emptying, and reduced defensive properties of the esophageal mucosa also contribute to reflux [36,37].

Accurate diagnosis of GERD can be challenging due to overlapping symptoms with other conditions [26,38,39]. Personalized treatment strategies tailored to individual patients are crucial, considering variations in symptom severity, treatment response, and complications. Acid suppression remains the mainstay of GERD treatment [40,41]. Factors such as diet, medications, obesity, smoking, and impaired mucosal barrier function also play a role [42,43]. Understanding these mechanisms is essential for effective management of GERD.

The commonly prescribed pharmacotherapeutic agents include: (1) PPIs are the first-choice treatment for GERD. Standard PPI therapy has shown high effectiveness, ranging from 90% to 100%, in individuals with mild symptoms of GERD. However, its efficacy decreases to around 60% in individuals with more severe disease. Research suggests that when it comes to maintenance therapy, low-dose PPI is equally effective compared to high-dose PPI. Nevertheless, although these drugs are effective, approximately 20–30% of patients experience an inadequate response and necessitate alternative medications. (2) Prokinetic drugs can be useful adjuncts in treating GERD by increasing the LES pressure, enhancing gastric emptying, or improving peristalsis. Clinically, however, these drugs are marginally useful. (3) Mucosal protective agents are less effective compared to antacids, alginates, H2RAs, and PPIs in treating erosive esophagitis and alleviating GERD symptoms. Their utility is limited when it comes to addressing duodenal and gastric ulcers. (4) Histamine and H2 receptor antagonists (H2RAs) demonstrate both safety and efficacy in managing symptoms of acute reflux disease.

### 2.2. Challenges in GERD Treatment

GERD management has a significant impact on individuals and healthcare resources. Managing individuals with GERD is estimated to be twice as expensive as treating symptoms in individuals without GERD due to the higher morbidity and increased costs associated with poorly managed GERD. Diagnosis and prognosis of GERD still require continuous improvement. Treatment options include lifestyle changes, PPI medication, and laparoscopic fundoplication. Emerging endoscopic and less invasive surgical procedures are also available. PPIs are commonly used but require ongoing long-term therapy monitoring for potential side effects [14]. Surgical intervention is considered as a last resort. Incorporating a fiber-rich diet can be beneficial in preventing and managing GERD, potentially improving quality of life [15].

The diagnosis of GERD does not have a universally recognized gold standard and typically relies on a combination of factors. These include assessing symptom presentation, conducting an endoscopic evaluation of the esophageal mucosa, reflux monitoring, and observing the response to therapeutic intervention. While heartburn and regurgitation are commonly associated with GERD, their sensitivity and specificity vary. A systematic review found that the sensitivity of heartburn and regurgitation for erosive esophagitis ranged from 30% to 76%, with specificity ranging from 62% to 96% [5]. Many guidelines suggest a trial of PPI therapy as a diagnostic “test” for patients with typical symptoms, assuming that a positive response to PPI treatment confirms the diagnosis of GERD. However, this approach has limitations. When endoscopy and pH monitoring are used as the reference standard, meta-analyses and prospective studies suggest a combined sensitivity of 78% and a specificity of only 54% [23].

PPIs reduce stomach acid and are commonly used for GERD, ulcers, and *H. pylori* infections. They are the first-line treatment for GERD, providing relief from heartburn. Antacids and alginate-based products offer alternative options. Long-term use of PPI carries risks such as infections, nutritional deficiencies, and interactions with other medications [23]. PPIs can impact oral health and alter the mouth’s microbiota [13]. Safety concerns and potential side effects include anemia, vitamin deficiencies, hypomagnesemia, and associations with conditions like kidney disease and gastric polyps. Most uncomplicated GERD cases improve within 4–8 weeks of treatment, but longer therapy may be necessary for refractory cases. The salivary oral microbial composition is important when studying GERD’s effects on the oral cavity [13]. Individuals diagnosed with GERD have a higher risk of developing periodontitis in comparison to those who do not have GERD [44].

NERD patients lack esophagitis but experience typical GERD symptoms and high esophageal acid exposure. PPI therapy has a lower response rate in NERD compared to erosive esophagitis. Refractory GERD may be attributed to reflux hypersensitivity and functional heartburn, requiring neuromodulation, psychological therapy, and complementary approaches [11,45]. Comprehensive evaluation, including symptom assessment and diagnostic tests, is needed to explore the diverse causes of the lack of response. Management strategies for refractory GERD involve alternative pharmacologic treatments and, if necessary, invasive anti-reflux options like laparoscopic anti-reflux surgery (LARS) or less invasive interventions like Transoral Incisionless Fundoplication (TIF), Transoral Incisionless Fundoplication (LINX), or Stretta. The approach should be tailored to each patient and risks and benefits should be considered [4].

GERD therapy follows a therapeutic ladder approach, progressing from less invasive to more invasive options. Natural orifice approaches like NOTES can be considered [46]. Novel diagnostic metrics, such as the post-reflux swallow-induced peristaltic wave (PSPW) index, baseline impedance, and mucosal impedance, show promise for establishing a clear GERD diagnosis [26]. Conventional barium esophagography and multimodality imaging play roles in GERD detection and assessment [10]. Comparative studies are lacking between the magnetic anti-reflux device Magnetic Sphincter Augmentation (MSA) and Nissen fundoplication. Still, MSA offers similar GERD control with the advantages of less bloating and a better ability to vomit and belch. However, it can cause more prolonged and severe dysphagia [32].

A multifaceted herbal medicine is potent in treating underlying causes of GERD and managing symptoms [47]. Traditional herbal medicines for GERD have been prevalent for a long time, but there is still limited clinical evidence supporting their effectiveness [48]. However, aloe vera has shown promise as a potentially safe and effective treatment option for reducing GERD symptoms [49].

### 2.3. Strategies for Relieving GERD

Accurate diagnosis of GERD and identification of complications require a thorough evaluation utilizing various diagnostic procedures [15,23]. The choice of diagnostic approach should be guided by the patient’s specific symptoms, medical history, and clinical presentation. A multidisciplinary approach involving gastroenterologists, radiologists, and other specialists is often necessary for optimal diagnostic accuracy and management of GERD [10,50]. Further research and advancements in diagnostic techniques are warranted to enhance diagnostic precision and improve patient outcomes in GERD: upper endoscopy, ambulatory acid (pH) probe test [39], X-ray of the upper digestive system [51], esophageal manometry [51], and transnasal esophagoscopy [52].

It is crucial to comprehend the molecular mechanisms involved in developing effective treatments for GERD. This revision concisely summarizes the guidelines, focusing on the sections concerning GERD treatment. The revision addresses important clinical issues such as (i) Treatment algorithms have been introduced to classify GERD into two categories: reflux esophagitis and non-erosive reflux disease, (ii) Treatment algorithms have been refined to address the varying degrees of severity in reflux esophagitis, and (iii) The utilization of vonoprazan has been recommended as part of GERD treatment strategies. The guidelines propose vonoprazan as the initial and maintenance treatment for severe reflux esophagitis. They suggest using either vonoprazan or PPI as the initial treatment for mild reflux esophagitis and both PPI and vonoprazan for maintenance treatment [33].

The ideal strategy for relieving GERD symptoms involves a combination of lifestyle changes, medications, and in some cases, surgical intervention:

1. Lifestyle changes include maintaining a healthy weight, as obesity contributes to GERD. Avoiding foods and drinks that trigger heartburn (like fatty or fried foods, alcohol, caffeine, and chocolate) can help. Avoiding late-night meals, elevating the head while sleeping, and quitting smoking can also reduce GERD symptoms.
2. Medication: Over-the-counter medications such as antacids, H2 blockers, and PPIs can help. A doctor may prescribe stronger doses of these or other medications for more severe cases.
3. Surgery: In severe cases, or when medication and lifestyle changes do not help, surgical procedures may be an option. The standard surgical treatment for GERD is a procedure known as Nissen fundoplication, which strengthens the lower esophageal sphincter, preventing acid reflux.
4. Regular follow-ups with a healthcare provider: GERD is a chronic condition, so regular monitoring by a healthcare provider is important to manage symptoms and monitor for any potential complications.

Proton-pump inhibitors (PPIs) represent a class of drugs most prominently known for their use in acid-related disorders [53]. They are often the first-line agents among gastroenterologists. PPIs, particularly rabeprazole sodium, are considered the most effective drug therapy for GERD [54]. However, rabeprazole sodium is less likely to interact with other drugs due to its minimal impact on the CYP2C19 genetic polymorphism [55,56,57]. Its physicochemical stability is challenging, leading to the formulation of enteric-coated tablets and delayed-release capsules. These formulations have a longer onset time, which may not be suitable for immediate therapeutic efficacy in GERD. PPI treatment affects the absorption of orally administered drugs by altering gastric pH and reducing gastric fluid secretion. The latest guidelines now recommend vonoprazan as the primary treatment option for both initial and maintenance therapy in cases of severe reflux esophagitis. For mild reflux esophagitis, the guidelines suggest using either PPIs or vonoprazan. These updated guidelines provide valuable clinical strategies to assist healthcare professionals in managing GERD patients effectively [33,41].

The development of drug delivery technology holds promise for revitalizing GERD medications, thereby enhancing their effectiveness and efficacy in treatment. Advancements in drug delivery technology have the potential to revolutionize the way GERD medications are administered and improve patient outcomes. Developing optimized drug formulations that can withstand the acidic gastric environment is crucial. These formulations aim to preserve drug stability, ensuring the medication remains effective upon reaching the intended site of action. Implementing advanced drug delivery technologies in GERD treatment can increase drug effectiveness, reduce side effects, and improve patient compliance.

### 2.4. Targeting Molecular Pathways

Gastroesophageal reflux disease (GERD) is characterized by the backward flow of stomach acid into the esophagus, causing symptoms such as heartburn, regurgitation, and chest pain. Several molecular pathways have been identified as potential targets for treating GERD. Here are some of the key pathways that researchers have focused on:

1. Acid secretion pathway

GERD development is closely linked to gastric acid production, which plays a central role. Proton Pump Inhibitors (PPIs) are commonly used to decrease acid production by inhibiting the H+/K+ ATPase pump in gastric parietal cells [58]. Acid reflux is a normal physiological phenomenon that occurs in everyone, but it can cause GERD symptoms when unsuppressed acid refluxes into the esophagus. Acid pockets are areas of unrestrained gastric acid that accumulate in the proximal stomach after meals and may serve as a reservoir for acid reflux, especially in a large hiatal hernia [58,59]. Acid pockets develop due to inadequate support from food to release acid in the proximal part of the stomach, leading to reflux into the esophagus.

**Figure 2:**
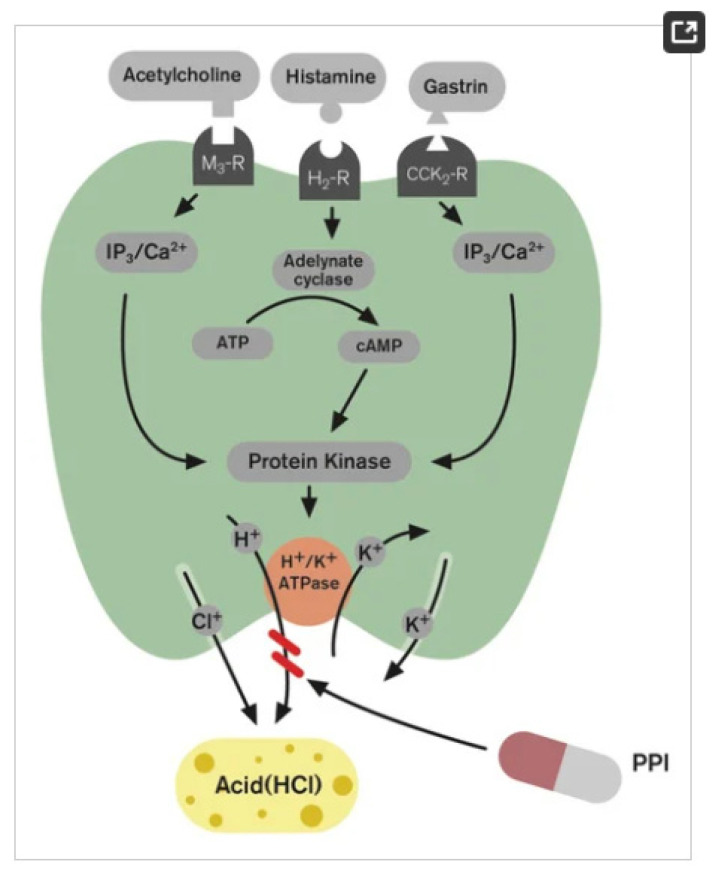
The stomach’s parietal cells have proton pumps called H+/K+ ATPase, which transport acid (H+) into the stomach lumen [61].

The main triggers for acid secretion in gastric cells are histamine, acetylcholine, and gastrin, to a lesser extent. Various molecules and receptors, such as ATP, cAMP, CCK2-R, H2-R, IP3, M3-R, and PPI, are involved in this process (Figure 2) [60].

Individuals with GERD have a higher prevalence of proximal pathological gastroesophageal reflux (PPGAP), characterized by longer length, higher position, and increased acid reflux likelihood [62]. Alginate-antacid rafts co-locate with the acid pocket and suppress postprandial acid reflux, indicating their potential as a targeted treatment [62].

New acid-suppressant drugs called PCABs act faster than PPIs, inhibit gastric H+K+ATPase, and are non-inferior in healing esophagitis [58,59]. P-CABs like vonoprazan and tegoprazan are prominent examples. Vonoprazan is effective in treating GERD and *H. pylori* infection and shows rapid onset and an extended half-life [63,64,65]. Intermittent use of P-CABs is preferred in managing reflux esophagitis [66]. Acid suppression is the primary therapy for erosive esophagitis (EE). Antacids provide rapid relief but lack sustained benefits, while alginates combined with antacids are more effective in relieving heartburn and acid reflux [67,68]. Research findings indicate that individuals with GERD exhibit a greater prevalence of proximal pathological gastroesophageal reflux (PPGAP) characterized by longer length, higher position, and an increased likelihood of acid reflux than those without GERD [69].

2. Esophageal motility pathway

Abnormal esophageal motility can contribute to GERD. Medications like prokinetic agents aim to improve lower esophageal sphincter (LES) tone and esophageal clearance to prevent reflux. GERD patients experience more and longer exposure to stomach acid. Strengthening esophageal muscle movement and protective properties can reduce reflux contact time and promote healing [4]. Prokinetics have limited effectiveness as a sole treatment for GERD and come with adverse effects. Some prokinetic drugs can potentially improve esophageal clearance and gastric emptying in GERD patients. However, limited evidence supports their effectiveness, and they carry risks of cardiac toxicity and neurological side effects. The 2018 US guidelines do not recommend using prokinetic drugs for patients with PPI-refractory GERD. Although prucalopride has demonstrated safety regarding cardiac issues, further clinical trials are needed to determine its efficacy in treating GERD [65]. In some cases, heartburn-like symptoms unrelated to reflux can be caused by psychological stress or esophageal muscle disorders, leading to a diagnosis of functional heartburn instead of GERD, even with negative results on symptom tests and PPI use [70].

Targeting transient lower esophageal sphincter relaxations (TLESRs) is a prominent focus in GERD therapy. Multiple receptors, such as GABAB, mGluR5, CB1, CCK, 5-HT4, muscarinic, and opioid receptors, initiate TLESRs [65]. Baclofen, which activates GABA receptors, shows promise in treating refractory GERD by inhibiting LES relaxation and preventing reflux [71]. Other agents like lesogaberan and arbaclofen placarbil have not shown significant effectiveness and were discontinued [72]. rGERD is caused by a weakened antireflux barrier, especially the LES, due to decreased LES pressure, hiatal hernia, and TLESR. Obesity, particularly central obesity, may contribute to rGERD by increasing gastric pressure [4].

3. Inflammation pathway

Chronic esophageal inflammation worsens GERD symptoms. Targeting inflammatory mediators like cytokines and chemokines may provide relief and reduce complications. Neuromodulators have limited efficacy for NERD. Visceral analgesics help manage symptoms in refractory PPI patients [4]. Increased epithelial permeability causes pain as nerves are exposed to the acidic contents, leading to tissue injury. The reflux of biliary material also damages the esophageal mucosa [73]. LPR-related mucosal disorders involve acid and pepsin exposure, triggering inflammatory responses [74]. Esophageal mucosal changes, inflammation, and nerve activity contribute to heartburn perception in GERD [75]. Acid affects acid-sensitive receptors, leading to neurogenic inflammation and pain [76].

4. Mucosal protection pathway

The esophageal mucosa acts as a protective barrier against acid exposure. Agents that strengthen mucosal defense mechanisms, like cytoprotective agents or mucosal enhancers, have potential therapeutic effects in GERD. Mucosal protective agents (MPAs) are commonly used alone or with PPIs to reduce symptoms effectively. PPIs can have side effects, including enteric infections. Refluxed gastric material damages the esophageal lining due to components like hydrochloric acid, pepsin, and duodenal juice, affecting epithelial cell junctions and increasing permeability [72]. The upper aerodigestive tract’s mucins, junctions, epithelial cells, and immune cells contribute to the protective barrier. Sucralfate and sucrose octasulfate (SOS) improve esophagitis. Sucralfate forms a thick gel layer, while SOS creates a protective film on the mucosal surface [74]. Using a mucoadhesive formulation with sodium hyaluronate and chondroitin sulfate shows promise in managing GERD, including in pediatric patients [77].

5. Sensory pathways

Esophageal hypersensitivity contributes to GERD symptoms. Modulating pain perception pathways, like TRP channels, offer new therapeutic approaches. Rome IV criteria classify reflux hypersensitivity (RH) and functional heartburn (FH) as esophageal functional disorders within the GERD spectrum. RH and FH are considered part of GERD only in the presence of abnormal esophageal acid exposure. Neuromodulators’ efficacy for RH and FH varies in clinical trials. Surgical therapy shows promise for RH, challenging the current classification. For pregnant patients with GERD, a step-up treatment approach involving lifestyle modifications, calcium-containing antacids, sucralfate, histamine-2 receptor antagonists, and PPIs can effectively manage symptoms [78,79].

### 2.5. Role of Nanoparticles in GERD Management

Controlled release technology revolutionizes the delivery of active substances, offering precise targeting and sustained release, thereby maximizing treatment efficacy while minimizing potential side effects. By harnessing the potential of controlled release technology, healthcare professionals can optimize therapeutic interventions, providing a promising avenue for improving patient care and achieving superior treatment outcomes [80]. NPs can also serve as carriers for drug delivery purposes [29]. They can be engineered to deliver medication to specific sites in the body, enhancing the drug’s effectiveness while minimizing side effects [29,81]. In the case of GERD, this could involve delivering drugs that neutralize stomach acid or strengthen the LES directly to the affected site. In this study, researchers synthesized nanoparticles loaded with drugs specifically for treating GERD, taking advantage of the advantages offered by a controlled release approach [80]. Some studies showed that these NPs can slow down the release of these drugs in acidic environments such as the stomach [82]. Enteric NPs, which can pass through the stomach to deliver drugs in the intestines, have been used for carrying things such as proteins and certain drugs, such as OMP and lansoprazole [82]. It is also important that the NPs can carry multiple drugs and fully protect them from the acidic stomach environment for better clinical results. However, making these NPs can be complicated and difficult on a large scale. This study looked at making easy-to-use, freeze-dried NPs of OMP for children and elderly patients who have trouble swallowing. They used a safe and biodegradable substance called CTS, which sticks to the mucus in the body, allowing the NPs to stay at the absorption site longer and improve drug absorption [83].

## 3. Chitosan-Based Nanoparticles

### 3.1. Chitosan-Based Nanoparticles in the Gastrointestinal Tract

Nanotechnology is an interdisciplinary field that combines principles from chemistry, engineering, physics, and biology. It involves synthesizing, characterizing, and utilizing nanoparticles (NPs) for a wide range of applications in science and technology. In recent years, nanotechnology has experienced rapid growth, driven by innovative techniques that enable precise control and generation of NPs [84,85].

Chitosan (CS) is a valuable biopolymer derived from chitin known for its biodegradability, biocompatibility, and low toxicity [86,87]. A significant barrier to its implementation is that it is only soluble in an acidic medium. The extensive amino and hydroxyl groups are the target groups for chemical changes to improve solubility NPs. The degree of deacetylation (DD) and molecular weight of CS have the greatest influence on its physical and chemical properties, including emulsification capacity, aggregation activity, rheological and solution, and physicochemical properties. In the gastrointestinal tract, CS has potential benefits, such as its ability to stick to mucosal surfaces [88,89,90], providing a protective layer over the esophageal lining, which could help protect against stomach acid in GERD [91]. It can also form a gel-like substance that acts as a barrier to prevent acid reflux. Additionally, CS can be used as a drug delivery system for medications that reduce stomach acid or improve the function of the lower esophageal sphincter [92,93].

### 3.2. Production and Characterization of Chitosan-Based Nanoparticles

A key focus in advancing nanotechnology is the development of safe, cost-effective, and environmentally friendly methods for synthesizing NPs. The main methods for producing CS NPs and nanocapsules (NCs) are ionic gelation, emulsification and crosslinking, complexation with polyelectrolytes, self-assembly, and drying processes [54]. The following subsections describe the most important methods for preparing CS NPs, also discussing recent improvements in the production schemes of conventional and novel CS NPs (such as optimized working parameters and conditions, new crosslinking agents, proper combinations of preparation schemes, etc.) [94]. Techniques like ionic gelation, self-assembly, and spray drying are favored as they align with promoting human health and sustainability [2]. The following table describes the most important methods for preparing CS NPs, also discussing recent improvements in the production schemes of conventional and novel CSNPs.

CSNPs have emerged as a promising area of research in nanomedicine and nanotechnology. These NPs offer several benefits and can be evaluated using various parameters, as depicted in Table 1. Characteristics and evaluation are needed to achieve consistent pharmacokinetics, enhance drug effectiveness, and improve patient outcomes in different therapies. However, it is crucial to emphasize that the design and optimization of nanoparticle-based drug delivery systems need careful attention to factors such as biocompatibility, stability, and manufacturing scalability to ensure their successful application in clinical settings and widespread adoption.

The characterization and evaluation of CSNPs provide valuable information about their physicochemical properties, safety, efficacy, and potential applications. This knowledge aids in the rational design, optimization, and translation of CSNPs for various biomedical and pharmaceutical purposes.

### 3.3. Functionalized Chitosan-Based Nanoparticles for Drug Delivery

CS is synthesized by deacetylating chitin, a polysaccharide in crustaceans’ exoskeletons and fungi’s cell walls. During the deacetylation process, the acetyl groups in chitin are removed, resulting in the formation of CS. Functionalized CS derivatives refer to modified forms of CS where various functional groups are introduced to enhance their properties and expand their potential applications [2]. The main objective of these modifications is to enhance CS’s solubility, expanding its potential applications [82].

**Figure 3:**
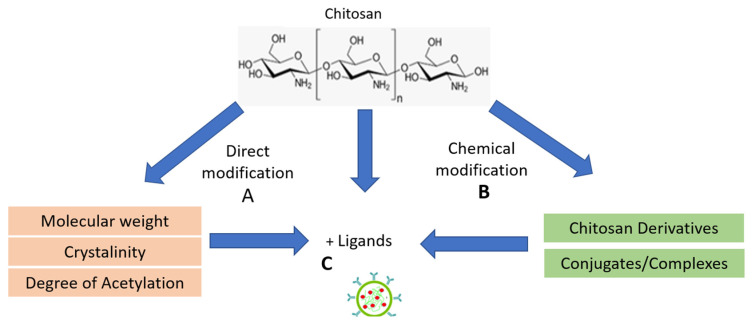
Schematic illustration showing the functionalization of CSNPs (A) Direct Modification, (B) Chemical modification and (C) Ligand Attachment [82].

1. Direct Modification (Figure 3A)

Modifying the molecular weight of CS involves altering its size and structure [122,123], which can be done through various methods such as enzymatic degradation, chemical processes, or physical treatments [124,125,126]. The resulting modified CS molecules may exhibit different properties and behaviors than the original CS, affecting their use in various applications. Low-molecular-weight CS is an attractive material with the potential for improving the absorption of poorly soluble drugs and proteins/peptides from the gastrointestinal tract because it can increase their membrane permeability [127].

Modifying the Degree of Deacetylation (DDA) of CS refers to changing the ratio of acetylated to deacetylated units in the CS molecule, which can be achieved through chemical or enzymatic processes [128,129]. Increasing the DDA results in more amino groups, affecting CS’s solubility, charge, and interactions. A positive charge of amino groups that may interact with negatively charged mucosal surfaces increases the mucoadhesive property [129]. This mucoadhesive property is very useful in gastrointestinal disorders.

Modifying CS’s crystallinity involves altering its molecular structure’s arrangement and organization, which can be achieved by controlling the drying process, using different solvents, or adding plasticizers. Changing the crystallinity can influence the mechanical strength, thermal stability, and other properties of CS-based materials. The mucoadhesive tendency of CS might also depend on its crystallinity [130].

2. Chemical modifications of chitosan (Figure 3B)

**Figure 4:**
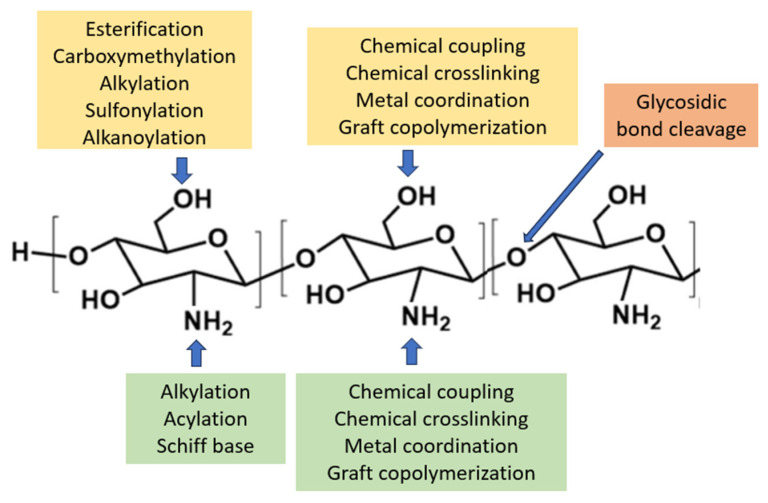
Chemical modifications of chitosan.

CS, a natural polysaccharide, has enormous potential in the biomedical field. The presence of the amino and hydroxyl groups of CS offers the opportunity of functionality using the esterification reaction, etherification reaction, and amide reaction toward diverse biotechnological needs, especially in drug delivery system applications (Figure 4).

a. Hydrophilic modification

CS’s limited water solubility hampers its biological uses. The solubility of CS can be modified by introducing additional functional groups into the polymer. To boost solubility, N,N,N-trimethyl CS (TMC) is made from N-methylation of N, N-dimethylchitosan, increasing quaternization [131]. For example, solubility in alkaline media can be achieved by introducing carboxyl groups into the CS polymer. Carboxyl groups have a p*K*_a_ value of ca. 4.5, indicating that all the carboxylic groups are expected to be deprotonated in pH ≥ 7, granting the carboxymethyl chitosan (CMChi) water solubility in neutral and alkaline pH [132].

b. Hydrophobic modification

Hydrophobization is usually carried out by introducing alkyl substituents of various lengths into the structure of CS due to reactions of its amino groups with fatty acids or their anhydrides (acylated derivatives) or by interaction with aldehydes followed by the reduction of azomethine bonds to secondary amines (alkylated derivatives) [133]. These hydrophobically modified CSs can create self-assembled nanoparticles to encase hydrophobic drugs in their core [134,135].

c. Amphiphilic modification

Hydrophilic (e.g., N,N,N-trimethyl, carboxymethyl, hydroxybutyl) and hydrophobic (e.g., cholesterol, deoxycholic acid) groups are connected to CS, resulting in amphiphilic-modified CS. Cholesterol-modified CS is synthesized by linking cholesterol 3-hemisuccinate to -NH_2_ using EDC. These amphiphilic molecules can carry small molecules, DNA, and proteins, forming nanoparticles through self-assembly. These nanocarriers offer a simple formation method and excellent biocompatibility [136]. Nanoparticle-constructed coacervates offer a favorable method for delivering drugs, holding the potential to handle and alleviate various gastrointestinal disorders effectively. This approach facilitates controlled drug release and prolonged presence within the gastrointestinal tract, addressing specific treatment needs [137].

3. Ligand (Figure 3C)

Adding ligands to CS involves attaching specific molecules, called ligands, to the CS molecule. Ligands are often chosen for their ability to bind to target molecules or receptors. This modification can enable CS to interact with specific cells, proteins, or compounds, enhancing its functionality in various applications. For instance, ligand-modified CS can be used in targeted drug delivery, where the ligand helps guide the CS-based carrier to a particular site in the body [138,139]. This approach allows for improved precision and efficiency in therapeutic applications.

### 3.4. Drug Release from Chitosan NPs

Drug release from the polymer matrix can be controlled through surface erosion, breaking of polymer bonds at the surface or bulk, and diffusion of the loaded drug, often using a combination of these procedures [140].

Controlled drug release from CSNPs is contingent upon several physicochemical attributes: shape, size, water absorption, degradation kinetics, chemical composition, molecular weight, solubility, and crystalline structure. Concurrently, drug-polymer interactions exert a substantial influence on the dynamics of drug release. In selecting manufacturing techniques, considerations are given to methods such as ionic gelation, polyelectrolyte complex, emulsion system, covalent cross-linking method, drying processes, supercritical assisted atomization, self-organized nanoparticles, and hydrogel, contingent on specific requisites [141].

CSNPs find utility in elevating drug bioavailability, orchestrating release kinetics, and facilitating the absorption profiles of hydrophilic drugs at specified target sites [142].

Drug release from CSNPs exhibits a distinctive biphasic pattern, characterized by an initial rapid release followed by a more gradual and controlled release phase. This biphasic behavior arises from a combination of factors, including the dissolution of adsorbed or trapped drugs from the particle surface, diffusion through the swollen polymer matrix, and polymer degradation or erosion, culminating in sustained drug release over an extended duration [143].

Mathematical models predict drug release kinetics from nanoparticles using equations that factor in nanoparticle properties, drug characteristics, and the environment. These models aid in understanding and designing controlled drug release for better treatment outcomes [86,144,145,146]. The Higuchi model is commonly used for diffusion-controlled releases, where cumulative drug release is proportional to the square root of time [147,148]. More complex models like Korsmeyer-Peppas or Weibull are often used to match better experimental data for different release mechanisms [149,150].

Mathematical models and preparation methods are crucial for effective drug delivery using CSNPs. Models predict drug release from CSNPs, while preparation methods shape nanoparticle properties affecting release [143]. Different methods yield varied sizes, shapes, and surface properties, affecting drug loading and release [144,151]. Models help predict release kinetics based on nanoparticle and drug properties. For uniform CSNPs, diffusion-based release is modeled, while porous nanoparticles involve diffusion and degradation. The interplay between models and methods tailors CSNPs for specific drug delivery goals, optimizing release profiles for desired therapeutic effects.

## 4. Chitosan-Based Nanoparticle-Based Systems for GERD Treatment

CS-based nanoparticles have been widely explored as drug delivery systems due to their unique biological properties, such as easy-to-create chemical modifications, biocompatibility, mucoadhesive feature, and absorption enhancement [136]. CS’s quaternization with GTMAC boosts water solubility via a positive charge [152]. CS’s synergy with medicine enhances pharmacological effects [153]. Additional benefits include improved bioavailability, targeted delivery, sustained release, and GI tract retention. CSNPs play a vital role in colon targeting [154]. CSNPs hold the potential for gastro retentive systems, curbing side effects via targeted drug release [155]. These advancements could enhance GERD treatment outcomes.

### 4.1. Acid Secretion Pathway

Treating upper GI disorders, including GERD, can be challenging and may involve various approaches like acid-reducing drugs, prokinetics, neuromodulators, herbal substances, psychological interventions, and alternative medicine [76]. However, effectively delivering drugs to the esophagus is difficult due to its short transit time and rapid clearance. Innovative drug delivery systems, such as controlled-release nanoparticles (CSNPs), aim to address this issue by enabling sustained drug release [156]. CSNPs can regulate acid levels through pH-responsive drug release mechanisms. They remain intact in the acidic stomach environment, protecting the drug, and undergo a pH-dependent transition in the less acidic esophageal area, releasing the drug where it’s needed (Table 2). CS is a promising material for formulating these drug delivery systems and enhancing oral delivery [157].

These findings highlighted nanoparticles’ potential to improve a drug’s therapeutic impact, offering a promising approach for treating acid-related conditions, including nocturnal acid problems. The sustained release and prolonged drug absorption provided by drug-loaded nanoparticles offer advantages over standard treatments, resulting in superior ulcer healing. Researchers have developed new compounds with extended durations of action to address the difficulties in managing GERD.

### 4.2. Esophageal Motility Pathway

The potential of nanoparticles to improve the therapeutic impact of a prokinetic drug offers a promising approach for enhancing esophageal motility (Table 3). The sustained release and prolonged drug absorption provided by drug-loaded nanoparticles offer advantages over standard treatments, resulting in improved esophageal motility.

The study elucidated the effects of crosslinking CSNPs on the erosion of swollen Nanofibers (NFs), their floating properties, drug release kinetics, and gastroprotective activity. The crosslinking process delayed swollen NF erosion, improved floating capability, and extended drug release, enhancing the drug’s gastroprotective efficacy. Additionally, CSNPs exhibited notable enhancements in drug bioavailability, bypassing hepatic metabolism and displaying improved brain targetability, which augments their potential to enhance patient convenience and compliance. The kinetics of drug release from CS microspheres were found to be best described by models originally developed for systems in which the release rate is primarily governed by the rate of diffusion through the matrix. These findings underscore the promise of CSNPs as a viable platform for optimizing drug delivery, providing a more efficient therapeutic approach for various medical conditions.

### 4.3. Inflammation Pathway

The use of nanoparticles to enhance the therapeutic efficacy of anti-inflammatory drugs shows great promise, offering a potential approach for relief and reducing complications associated with inflammation. The sustained release and prolonged drug absorption achieved through drug-loaded nanoparticles present several advantages over conventional treatments, improving heartburn relief and reducing associated complications. Kuadkaew et al. have shown that curcumin suspended in CS dissolves in acetic acid. Inhibiting the expression of COX-2 may also delay the healing of NSAID-induced gastric ulcers [168].

### 4.4. Mucosal Protection Pathway

Mucosal protective agents (MPAs) are frequently employed alone or in conjunction with Proton Pump Inhibitors (PPIs) to alleviate symptoms effectively. They work by forming a protective film on the mucosal surface, offering a promising approach for managing GERD (Table 4). This protective layer helps shield the mucosa from the damaging effects of stomach acid, reducing inflammation and discomfort associated with GERD.

### 4.5. Sensory Pathways

Recent studies on Non-Erosive Reflux Disease (NERD) have revealed the importance of visceral hypersensitivity (VH). VH refers to heightened sensitivity of the viscera to painful stimuli or negative reactions to normal physiological stimuli due to a decreased pain threshold. VH in NERD involves abnormal neurotransmitters, acid-sensitive receptor activation, and psychological factors. Substance P (SP) and calcitonin gene-related peptide (CGRP) are key neurotransmitters in pain signal transduction, playing a significant role in VH. VH contributes to acid reflux and heartburn symptoms in NERD patients. Treatments using TRPV1 antagonists, tricyclic antidepressants, and other drugs have shown good results in managing NERD [171]. Among various oral delivery approaches, CSNPs are promising vehicles with the potential to enhance oral drug retention and controlled absorption. They hold promise for treating local diseases within the gastrointestinal (GI) tract and systemic diseases. However, specific to sensory pathways, a combination has not yet been found with CSNPs in GERD therapy.

### 4.6. Combination Therapies

Combination therapies are gaining attention as a strategy to enhance therapeutic outcomes and address the limitations of single-agent treatments [172]. The “drug atlas” approach introduced by Narayan et al. identifies novel synergistic combination therapies [173]. Utilizing multiple drugs to target multiple pathways has emerged as a promising alternative with improved effectiveness and lower toxicity than single-drug treatments [88]. Computational methods, particularly machine learning, offer valuable strategies to predict effective drug combinations and overcome drug resistance [172]. Biomaterials, particularly CS, have advanced for controlled drug release to treat infections like *H. pylori*. Researchers are developing “smart” biomaterials that respond to environmental stimuli for on-demand drug delivery. Emerging technologies, including bionic drug delivery systems, phage therapies, and metallic biomaterials, show promise but require further dosage, toxicity, and treatment duration research. Probiotic composites for eradicating *H. pylori* also need more clarity on optimal use. Metal nanoparticles (NPs) like silver and zinc are gaining interest for their antibacterial effects against *H. pylori*, working at low concentrations to reduce the risk of drug resistance [174].

The use of drugs in combination can be divided into two approaches: some are combined within a single formulation (Table 5), while others involve separate formulations. The only difference lies in the efficiency of usage; both approaches offer favorable impacts in terms of their effectiveness.

A meta-analysis involving 16 studies and 1446 participants compared the effectiveness of PPI plus prokinetics treatment against PPI monotherapy for GERD symptoms. The combination treatment showed a significant reduction in symptoms regardless of the prokinetic type, refractoriness, or ethnicity. Treatment with PPI plus prokinetics for at least 4 weeks was more beneficial than PPI monotherapy for overall symptom improvement. However, the quality-of-life scores did not show improvement with the combination therapy. Adverse events were similar between the two treatment groups. Another analysis of 11 studies with 841 participants focused on PPI plus domperidone treatment and found a significant reduction in GERD symptoms compared to PPI monotherapy [177]. Adverse events were comparable between the two groups. Combining a prokinetic agent like domperidone with a PPI proved safe and effective in treating GERD. However, another study suggested that there may be no additional benefit of combining PPIs with prokinetics compared to PPI monotherapy in adult patients with overlapping functional dyspepsia and GERD, suggesting that PPI monotherapy alone may be sufficient as an initial treatment option [178,179]. Combining prokinetic agents and PPIs has been shown to improve GERD symptoms in individuals with high scores on the FSSG scale [180].

Using additional therapies alongside PPIs has shown positive results in some studies. One approach combines mucosal protective agents and acid inhibitors, which have effectively controlled symptoms and healed mucosal lesions. Switching to vonoprazan 20 mg per day for patients who do not respond well to 8-week PPI treatment has shown improved symptom control and faster healing. However, these studies were not controlled [12].

Combining patients with partial response to PPIs with mucosal protective drugs like alginate, hyaluronic acid, chondroitin sulfate formulations, or bile acid sequestrants can provide significant benefits. On the other hand, combining mosapride with a standard dose of PPI for four weeks did not show better results than using PPI alone in patients with PPI-refractory GERD [181].

There is limited evidence comparing ranitidine and omeprazole use in infants and children. However, a study focusing on infants found that both medications had similar effectiveness, although using a higher ranitidine dose may be more beneficial. Omeprazole will likely provide better relief from symptoms than ranitidine [182].

Combining rabeprazole and sulpiride has reliably improved respiratory function and psychoemotional status, and reduced the clinical and endoscopic aspects of GERD. This combination and bile acid therapy appear to be effective [183].

A study comparing different treatment groups showed significant response rates after one week and one month of treatment. However, the group receiving “lansoprazole plus metoclopramide” had a significantly higher response rate than the “ranitidine plus metoclopramide” group. Combining either acid suppressant with metoclopramide resulted in a higher response rate than using only one medication before intervention [184]. However, combining mucolytics with PPIs did not lead to faster or more effective relief of symptoms in patients with LPR than using PPIs alone [185].

## 5. Perspective

GERD is a common stomach condition that is difficult to diagnose and treat [12]. According to guidelines, patients with typical symptoms should initially use a PPI. If reflux symptoms continue after 8 weeks of PPI treatment, an esophagus endoscopy is recommended. During the procedure, biopsies are taken to rule out the presence of eosinophilic esophagitis [186]. GERD diagnosis involves endoscopy and pH testing to consider similar conditions. It can be clinically diagnosed based on symptoms like heartburn, physiologically through abnormal pH levels, anatomically by observing esophagitis during endoscopy, or functionally based on the antacid response. However, the correlation between these approaches is weak, necessitating the development of comprehensive metrics. New metrics like the PSPW index, baseline impedance, and mucosal impedance show promise in aiding diagnosis. A catheter-based balloon with sensors used during endoscopy has shown the potential to differentiate GERD from EoE [23]. Genetic testing plays a role in determining treatment for complex cases of GERD [12]. When GERD does not respond to PPIs, objective testing is required, and management strategies may involve genetic testing, medical therapy, or surgery/endoscopy.

The development of PPI treatments that are safer and more effective is focused on improving GERD treatment, especially for resistant cases [12]. Clear guidelines are necessary to ensure the appropriate and well-managed use of PPIs, including duration and regular check-ups, to address the global issue of misuse and overuse [61]. The ongoing advancements in PPI drugs, such as vonoprazan, provide alternatives for individuals who do not respond well to traditional PPIs [12]. The successful implementation of ARET relies on careful patient selection and a comprehensive understanding of the underlying mechanisms associated with each treatment option [187].

Ongoing advancements characterize the future of GERD treatment. Previous research about using collagen in 1988 has paved the way for further developments [24]. These coatings, incorporating Eudragit RS100 as the core and Eudragit S100 and hydroxypropyl methylcellulose phthalate HP55 as enteric coatings, effectively retain gastric acid and provide a gradual release of lansoprazole [188]. Addressing *H. pylori*, a major contributor to gastritis and recurrent duodenal ulcers, remains a focus. Gastro-retentive drug delivery systems (GRDDS), such as buoyant, mucoadhesive, and dual-working systems, have been developed to improve the effectiveness of oral medications. Mucoadhesive polymeric oral drug delivery systems show promise for improving the effectiveness of oral medications, especially those sensitive to stomach acid [174]. An in situ floating system has been developed for sustained delivery of Esomeprazole, a drug used for peptic ulcer diseases [189,190]. The EsoCap delivery system uses a small capsule with a medicine-loaded film that is mucoadhesive [191]. GADA is a new drug delivery mechanism made of β-Glucan and Docosahexaenoic Acid (DHA). GADA targets DHA transporters and receptors in the GI tract, effectively delivering hydrophobic drugs and remaining stable for over 12 h [192]. Orally disintegrating tablets (ODTs) can potentially improve treatment for GERD patients, especially those with swallowing difficulties like the elderly and pediatric patients [193]. There is growing interest in developing outpatient endoluminal therapies as promising options for GERD treatment [65].

In the future, it is crucial to explore alternative approaches, such as nanotechnology, to improve the management of GERD. Nanotechnology has great potential in the field in the future. Nanoparticle-based therapies, especially those using CS, aim to increase the effectiveness of PPIs and other drugs by targeting the esophagus, prolonging drug release, and reducing dosing requirements. Extensive and comprehensive research is required to fully understand the characteristics and safety of CS-based nanoparticle treatments for GERD. The vision for the future also involves rigorous testing to assess factors such as drug distribution, processing speed, and long-term effects. Surface modifications and careful material selection play a crucial role in ensuring the safety and efficacy of nanoparticles in their interaction with cells. The ongoing development of GERD treatments signifies a promising future with improved patient therapeutic options. Nanoparticles like silver NPs, zinc oxide NPs (ZnO NPs), magnetic NPs, and pH-sensitive gold NPs have demonstrated inhibitory effects on *H. pylori*, targeting its respiratory system, biofilm formation, urease enzyme, or employing thermal treatment [174].

In shaping the future, it is important to prioritize non-medical interventions for managing GERD. Lifestyle modifications play a crucial role. Incorporating a high-fiber diet, reducing salt intake, and engaging in regular exercise are key to improving GERD symptoms [3]. Looking ahead, healthcare providers should emphasize the significance of these lifestyle changes in the management of GERD [3].

The main components of GERD management include a combination of medications and lifestyle modifications [71]. A comprehensive evaluation of dietary and lifestyle factors and proper timing and dosage of PPIs form the initial approach for GERD management. Subsequent interventions should be tailored based on diagnostic tests like EGD, HREM, MII-pH monitoring, and gastric emptying tests [4]. Future developments encompass diagnostic advancements, minimally invasive surgical technologies, and acid-reducing drugs that protect the esophagus. The use of NPs in drug delivery systems shows promise in addressing GERD treatment challenges [194]. Collaborative precision medicine is set to shape the future of minimally invasive GERD treatments, surpassing technological advancements [195].

## 6. Conclusions

GERD is a common disorder, complex to diagnose and treat, and requires a clear definition because its symptoms can overlap with other esophageal problems. Treatment includes medications such as PPIs and lifestyle changes. Diagnosis will continue to develop as well as PPI—usually used first-line drugs will continue to experience development to improve weaknesses of previous drugs. Improvement of drug weaknesses can be done by using CS-based NPs. The future of CS-based NPs in treating GERD looks promising, with the potential for better treatment outcomes, customized solutions, and better patient care.

## Figures and Tables

**Table 1:** Advantages and limitations of CS preparation.

No	Preparation Method	Principle	Characteristics	Advantages	Limitations	Ref.
1	Covalent cross-linking method	The crosslinking reaction between the aldehyde group and amine is a covalent interaction that forms an imine bond and an acetal bond with a hydroxyl group.	This method generated a more compact surface with a smoother surface, producing NPs with sizes 100–400 μm.	The cross-linking agent makes it more stable against changes in pH, temperature, and damage, including biological and mechanical factors.	Commonly used agents to strengthen CS are epoxides and aldehydes, like glutaraldehyde (which can be toxic and need safer options to toughen CS.)	[95,96,97]
2	Ionic gelation	The electrostatic interactions between the polyanion (negative charge) bonds to an amino group (positive charge).	This process produces spherical particles within a targeted size range of 67 to 690 μm. The size is controlled by the amounts of CS and TPP, which depend on factors such as the degree of acetylation, molecular weight, viscosity, concentration, and the ratio of CS to TPP, pH, salts, ionic strength, temperature, stirring speed, the rate of TPP addition, and purification methods.	Easy process to conduct, facilitates low molecular weight drugs. Avoid the use of organic solvents and high temperatures.	Limited control over particle size and distribution leads to stability issues over time. Polyelectrolytes affect the loading capacity for hydrophobic and high molecular-weight drugs. Batch-to-batch variability poses challenges for scale-up. Additionally, the encapsulation efficiency of TPP/CS nanoparticles is limited, and they tend to have poor mechanical strength.	[98,99,100,101,102]
3	Polyelectrolyte complex (PEC)	The ionic interactions with negatively charged polymers result in self-assembly or the formation of strong reversible electrostatic bonds in self-assembly systems.	Factors like polyelectrolyte molecular weight, polymer chain flexibility, reaction temperature, ionic strength, and pH of the medium influence stability. NPs can be of varying sizes from 50 to 700 nm.	Polymer chains directly interact, forming networks that eliminate the need for crosslinking agents. Simple preparations and high drug-loading efficiency. The reaction is typically performed in aqueous solution without catalysts or initiators. The resulting macro-PECs are amorphous.	The complexity of process optimization, batch-to-batch variability (scale-up challenges), influence of polyelectrolyte, and stability challenges.	[89,95,99,103,104]
4	Emulsion Droplet Coalescence (Emulsion Crosslinking and Precipitation)	CS aqueous solution is emulsified in the oil phase and stabilized with a surfactant to form stable emulsion droplets, then crosslinked using a suitable cross-linking agent.	The size of NPs depends on the cross-linking agent amount and emulsion stirring speed.	Strong cross-linking agents are typically employed to achieve better control over particle size.	Emulsion cross-linking has drawbacks: complex procedures, harsh solvents, and incomplete removal of unreacted cross-linking agents, which can harm protein stability.	[94,105]
5	Emulsification Solvent Diffusion	The partial miscibility of an organic solvent as an oil phase with water.	Increasing the concentration of a water-miscible solvent makes it possible to reduce particle size.	This method works for hydrophobic and hydrophilic drugs. For hydrophilic drugs, a double emulsion is formed with the drug dissolved in the inner aqueous phase.	This method’s drawbacks include using organic solvents and high shear forces during nanoparticle preparation.	[106,107]
6	Emulsification Solvent Evaporation	The encapsulation of a drug within the water-insoluble polymer.	The emulsion is prepared with a suitable external phase based on the polymer and drug used, followed by solvent evaporation to form nanospheres. Size range 100–300 nm	Solvent evaporation precipitates CS nanoparticles by removing the solvent from a CS solution based on their solubility difference.	Using harsh solvents and incomplete removal of unreacted cross-linking agents.	[106,107]
7	Reverse Micelles	Surfactant in organic solvent forms reverse micelles. Aqueous CS and drug solution are added while vortexing to maintain clarity.	This technique offers a narrow size distribution of less than 100 nm.	Particle size can be controlled by adjusting the cross-linking amount. It enables the entrapment of insoluble compounds, enhancing compound release, bioavailability, and efficacy at lower doses.	Drawbacks include organic solvent use, time-consuming preparation, and complex washing steps.	[108,109,110]
8	Spray Drying	The use of a flux of hot air to dry spray drops	The particles are smooth, round, and uniform, ideal for stable drug delivery. The attachment of additional components enhances stability and controlled drug release. Particle size depends on spray speed, crosslinking strength, and airflow rate, ranging from 166–1230 nm.	These methods are quick, consistent, and fit various materials. They can make particles of different sizes that stay stable. It works well for polymers, nanoparticles, and nanocomposites.	The process produces large particles and their distribution. The process requires a sophisticated and intricate apparatus system.	[99,111,112]
9	Supercritical Fluid Drying	High-pressure CO_2_ mixed with acidic water dissolves CS, then sprayed into hot air. CO_2_, acetic acid, and ethanol induce CS crystallization via a supercritical antisolvent process.	This process made CSNPs in the range of 8.15 to 400 nm in size. The molecule’s weight affects the particles’ size, structure, and stability.	Supercritical CO_2_ is eco-friendly, reduces harmful solvents, and enables the production of small particles. It is suitable for large-scale use and offers sustainability benefits. It is particularly useful for compounds with poor solubility in water.	The process requires a sophisticated and intricate apparatus system.	[113,114,115]
10	Electrospraying Technique	Electrospraying is a method that uses electrical force to convert a liquid into solid nanoparticles.	Electrospraying produced thermally stable nanoparticles with a 100–483 nm size range. These nanoparticles exhibited higher encapsulation efficiency, particle stability, and a highly positive charge.	Electrospraying is a cost-effective and simple method for producing nano- and micro-particles used in drug delivery and pharmaceutical applications.	NPs exhibit non-homogenous and irregular shapes, with a higher tendency to form small agglomerates during drying.	[107,116,117]
11	Precipitation/coacervation	The Marangoni effect is utilized to obtain a colloidal suspension of nanoparticles by slowly adding the oil phase to the aqueous phase with moderate stirring.	Nanoprecipitation rapidly mixes a CS solution with a non-solvent, resulting in nanoparticle precipitation. It allows for the synthesis of nanoparticles with narrow size distribution and without shearing stress. Key parameters like the organic phase injection rate, aqueous phase agitation rate, and oil phase/aqueous phase ratio significantly affect the fabrication process. Size in range 50 to 300 nm.	This method is used mostly for hydrophobic drug entrapment but is sometimes employed to incorporate hydrophilic drugs. This technique yields highly reproducible nanoparticles.	Organic solvent use, time-consuming preparation, and complex washing steps.	[118,119]
12	Microfluidic Method	An integrated microfluidic device with three stages (nucleation, growth, and separation) was developed for precise and controllable mixing.	Promising platforms for fabricating CS-based NPs with monodisperse size distribution, controlled morphology, and microstructures include 75–105 nm particles with low polydispersity (0.15–0.22) and positive zeta potentials (6–17 mV).	Reproducible and cost-effective production of CS-based NPs enables the safe delivery of hydrophilic biotechnological drugs like proteins and nucleic acids. The size limit of approximately 100 nm optimizes therapeutic activity and facilitates scale-up for industrial production.	The process requires a sophisticated and intricate apparatus system.	[120,121]

**Table 2:** CSNPs in the Acid Secretion Pathway.

No	Drug	Chitosan Modification	Preparation	Result	Ref.
1	Lansoprazole	CS with TPP solution	Ionotropic gelation method	In-vitro release of drug follows zero-order and showed sustained release behavior for a period of 24 h.	[158]
2	Lansoprazole	Freeze-dried CS/poly-(glutamic acid)	Samples were freeze-dried and filled in an enteric-coated capsule	The formulation showed good swelling properties. Drug encapsulation efficiency of formulation F3 was 82.82%, and in vitro, the release of prepared formulation F3 was 94% after 8 h of dissolution in 7.4 pH phosphate buffer. FTIR and DSC studies showed no interaction between the drug and the polymer.	[159]
3	Lansoprazole	Thiolated CS microspheres for mucoadhesion	Emulsifying method uses liquid paraffin light and heavy in a ratio of 50:50 as a dispersing medium and glutaraldehyde as a cross-linking agent.	The release profiles showed first-order release behavior up to 12 h, where the highest drug release was 88.89% of the lansoprazole loaded in the thiolated CS microspheres, indicating a strong crosslinking between CS and glutaraldehyde.	[92]
4	Omeprazole	Omeprazole entrapped and tetrathiomolybdate adsorbed CS nanoparticles CSNPs-OME-ATM		An enhancement of fluorescence of Nile red added to CSNPS@OME@ATM at pH 2.5 and 6.5 indicates the release of H2S, an essential trigger for ulcer healing. Nitrite levels (1.37 mmol/g), catalase activity (33.68 mmol H_2_O_2_/min/mg protein), decreased gastric juice content, and histopathological evaluation confirmed the protective and ulcer-healing nature. The study confirmed that CSNPs with omeprazole and tetrathiomolybdate have superior protection and healing of gastric ulcers.	[160]
5	Omeprazole (OMP)	Eudragit L 100-55/CS enteric nanoparticles	The nanoparticles were formed by complex coacervation method using CS and Eudragit L100/55 (EU)	in vitro release studies showed the pH sensitivity of nanoparticles and OMP release was pH-dependent. In vivo, the pharmacological assessment revealed that the optimized formulation could protect rat stomachs against ulcer formation induced by indomethacin compared to the group that received normal saline, which demonstrated severe peptic ulcer and hemorrhagic spots.	
6	Omeprazole	Omeprazole Mucoadhesive tablets were prepared using the direct compression Method. The drug, polymers, and excipients were mixed homogeneously in a glass mortar for 20 min. The powder blend was then screened through sieve no # 80. The mixture was then compressed using an 8 mm biconcave punch in a single stroke using 8 stations rotary machine	Formulations were prepared with CS as the primary polymer and Carbopol 934, Hydroxy Propyl Methyl Cellulose (HPMC K4M), and Xanthan gum as a secondary polymer.	The release studies indicated that the prepared Omeprazole mucoadhesive tablets improved the bioavailability by avoiding first-pass metabolism. The in vitro studies have shown that this is a potential drug delivery system for Omeprazole with a considerably good stability and release profile.	[161]
7	Omeprazole	The Gum Arabic (GA)—O-carboxymethyl CS (OCMC) microcapsules—OCMC LbL	Prepared by layer-by-layer (LbL) assembly and genipin crosslinking	Pharmacokinetic analysis indicated that entrapment by GA—OCMC LbL assembly greatly improved the bioavailability of omeprazole, and crosslinking by 0.1 mg/mL genipin led to the highest value of 8.76 relative to the control formulation. It was concluded that the GA—OCMC LbL microcapsules could be used for the oral delivery of nutraceuticals, and its delivery performance could be tailored by varying the genipin crosslinking degree.	[162]
8	Famotidine	Eggshell membranes (ESMs) and CS		These nanoparticles demonstrated a controlled release strategy, improving the targeted delivery of FTD for GERD treatment.	[80]
9	Famotidine	Montmorillonite-Famotidine/CS Bio-nanocomposite Hydrogels as	Prepared using an ion exchange process	The optimized MMT-famotidine (FMT)/CH bio-nanocomposite hydrogels displayed a controllable and sustainable drug release profile with suitable mucoadhesion and prolonged retention time in the stomach. Thus, the results demonstrated that the fabricated mucoadhesive bio-nanocomposite hydrogels could remarkably increase the therapeutic efficacy and bioavailability of FMT by the oral route.	[163]

**Table 3:** CSNPs in the esophageal motility pathway.

No	Drug	Chitosan Modification	Preparation	Result	Ref.
1	Nizatidine	Glutaraldehyde-crosslinked CS-polyethylene oxide nanofibers as a potential gastro retentive delivery system	Glutaraldehyde-crosslinked	The crosslinking delayed the swollen NFs erosion, enhanced their floating, extended the drug release, potentiated the gastroprotective activity of NIZ, and maintained the normal gastric wall architecture, COX-2 expression, and the gastric tissue content of the oxidative stress markers.	[144]
2	Metoclopramide hydrochloride	CS and chondroitin sulfate microspheres	Formaldehyde as cross-linker	CS microspheres prepared with more than 15% formaldehyde (*w*/*w* concerning polymer) showed good control release (more than 8 h), and medium pH did not affect release rates. Release from CS microspheres prepared with 20% formaldehyde was independent of pH, suggesting this may be the most appropriate formulation.	[164]
3	Metoclopramide	5-methyl pyrrolidinone CS (MPC) mucoadhesive microparticles for the nasal administration of drugs	The ionically crosslinked hydrogel was hypothesized	The hydrogel formation from microspheres was studied in different media and pHs. Microspheres can control the in-vitro MC release. MPC microparticles show good in-vitro mucoadhesive properties and ex-vivo-controlled permeation profiles. The hydrogel formation depends mainly on the medium used: ionically crosslinked hydrogel was hypothesized. These in-vitro and ex-vivo preliminary results show that spray-dried microspheres based on MPC could be a suitable nasal delivery system for administering metoclopramide.	[165]
4	Metoclopramide hydrochloride; MTC	A PEGylated Tween 80–functionalized CS–lipidic (PEG-T-Chito-Lip) nano-vesicular hybrid	The blank (MTC-free) lipidic nanovesicles, CS-lipidic nanovesicles, PEGylated CS-lipidic nanovesicles, Tween 80–functionalized CS-lipidic nanovesicles, and PEGylated Tween 80–functionalized CS–lipidic (PEG-T-Chito-Lip) nanovesicles (in addition to the MTC–loaded nano-vesicular preparations) were created.	The performance of the dual-optimized PEG-T-Chito-Lip nano-vesicular hybrids for intranasal administration evidenced MTC-improved bioavailability, circumvented hepatic metabolism. It enhanced brain targetability, with increased potentiality in heightening patient convenience and compliance.	[93]
5	Metoclopramide	Mucoadhesive polymer CS	The CS microspheres were prepared by simple emulsification phase separation technique using glutaraldehyde as a crosslinking agent.	Drug release was diffusion controlled and followed non-Fickian diffusion. Microspheres prepared using polymer to drug ratio of 4:1 were suitable for oral controlled release with good mucoadhesion up to 8 h. The microspheres exhibited a good swelling index and 72% drug entrapment efficiency.	[166]
6	Mosapride Citrate	Intranasal Surface-Modified Mosapride Citrate-Loaded Nanostructured Lipid Carriers (MOS-SMNLCs) Surface modification using CS was applied.		Pharmacokinetic studies showed a 2.44-fold rise in bioavailability compared to MOS suspension and 4.54-fold compared to the orally marketed product. In vitro/in vivo studies have proven a correlation between in vitro permeation through sheep nasal mucosa and in vivo absorption.	[167]

**Table 4:** CSNPs in the mucosal protection pathway.

No	Drug	Chitosan Modification	Preparation	Result	Ref.
1	Zuojin Pill (ZJP), a traditional Chinese medicine formula, consists of *Coptis chinensis* Franch. And *Evodia rutaecarpa* (Juss.) Benth	CS and alginate	Internal gelation of alginate with ion (Ca^2+^) and coating CS outside.	Mucoadhesive microspheres loaded with alkaloids were prepared using CS and alginate. They exhibited good release properties and adhered well to the gastric mucosa. In an ethanol-induced gastric mucosal injury model in rats, these mucoadhesive alkaloid-loaded microspheres reduced TNF-α and IL-1β production, downregulated iNOS, TNF-α, and IL-1β mRNA expression. They increased the production of gastroprotective factor PGE2 in the gastric mucosa.	[169]
2	*Morus alba* L. Extract	*Morus alba* L. ExtractLoaded CS Microspheres	CS was dissolved in 10 mL of 5% aqueous acetic acid. The extract solution was added and emulsified with light liquid paraffin (100 mL) using a magnetic stirrer at various rates for 5 min. Glutaraldehyde (GA) was added in different ratios as a cross-linking agent, and the mixture was stirred for 2 h.	Histopathology of tissue sections also confirmed the protection of gastric mucosa on pretreatment with MEM at 500 mg/kg p.o. Based on these findings, we can conclude that prepared microspheres can be used to develop a sustained-release formulation of extract to manage gastric ulcers.	[151]
3	Citrus-apple pec-tin	Citrus-apple pectin and CS.	The gel was prepared in two stages: first, using methylcellulose and citrus-apple pectin, and second, with CS. To speed up the gelation process, the mixture was cooled to 5–10 °C.	The tested gels have adhesive properties that allow them to remain on the esophageal mucosa for an extended period, protecting against the harmful effects of gastric or bile contents. These gels have a wide pH range, allowing for selecting the optimal pH to suit the esophagus based on the reflux type.	[170]

**Table 5:** Combination Therapies in one drug delivery.

No	Drug	Chitosan Modification	Preparation	Result	Ref.
1	Omeprazole (OMP) and curcumin (CURC)	A pharmaceutical dosage form containing omeprazole (OMP) and curcumin (CURC) to treat experimental peptic ulcers. OMP and CURC were preliminarily complexed with hydroxypropyl-β-cyclodextrin to enhance their solubilization. After that, the combined complex (CURC/OMP) was loaded to alginate beads to sustain their release and then coated with CS.	Algininat-CS complex	The OMP/CURC beads showed a more stable particle size (0.52 ± 0.01 mm) after 6 weeks. In conclusion, the OMP/CURC hydrogel beads give stronger anti-ulcer effectiveness than free OMP, CURC-only beads, and OMP-only-loaded beads, indicating a prospective application for managing peptic ulcers.	[175]
2	Amoxicillin, clarithromycin, and omeprazole	Targeted sustained-release nanoparticles of CS–glutamic acid conjugates	The CS–glutamate nanoparticles were prepared by using the ionotropic gelation method.	In vitro, the antibacterial efficacy of optimized formulations containing monotherapy and triple therapy on isolated cultures of *H. pylori* was assessed. In vivo, clearance studies and histopathological studies were also carried out on Swiss albino mice to evaluate the efficacy of triple therapy containing a targeted nanosystem for the treatment of *H. pylori*.	[176]

## Data Availability

Not applicable.
